# The phosphorylation status of NONPHOTOTROPIC HYPOCOTYL3 affects phot2-dependent phototropism in *Arabidopsis*

**DOI:** 10.1080/15592324.2022.2027138

**Published:** 2022-01-23

**Authors:** Taro Kimura, Ken Haga, Tatsuya Sakai

**Affiliations:** a Graduate School of Science and Technology, Niigata University, Niigata, Japan; b Department of Applied Chemistry, Nippon Institute of Technology, Saitama, Japan

**Keywords:** *Arabidopsis*, phototropism, phot2, NPH3, protein phosphorylation

## Abstract

The blue light photoreceptors, phototropin 1 (phot1) and phot2, and their signal transducer, NONPHOTOTROPIC HYPOCOTYL3 (NPH3), are activators of the phototropic responses of *Arabidopsis* hypocotyls. In a recent study, we reported that the control of NPH3 phosphorylation at serine 7 (S7: or S5), S213, S223, S237, S467, S474 (or S476), and S722 (or S723) contributes to the photosensory adaptation of phot1 signaling during the phototropic response. Phosphomimetic NPH3^SE^ mutant and unphosphorylatable NPH3^SA^ mutant on those serine residues function efficiently under blue light conditions at fluence rates of 10^−5^ µmol m^–2^ s^–1^ and 10^–3^ µmol m^–2^ s^–1^ or more, respectively. We here demonstrate that phosphomimetic NPH3^SE^, but not unphosphorylatable NPH3^SA^, promotes phot2-dependent phototropism under blue light condition at 100 µmol m^–2^ s^–1^. This result suggests that phot1 negatively controls phot2 signaling through the dephosphorylation of NPH3 at those residues and that the hyperactivation of phot1- and phot2-NPH3 complexes does not occur at the same time under high intensity blue light. We hypothesize that the dephosphorylation of NPH3 on those serine residues suppresses both phot1 and phot2 signaling, which results in different impacts on phot1- and phot2-dependent hypocotyl phototropism due to the differences in the photosensitivity and activation levels of phot1 and phot2.

The phototropic responses in plants manifest as changes in the growth direction of plant organs in response to the direction of blue light irradiation.^1^ The blue light photoreceptors, the phototropins, are responsible for the induction of the phototropic responses and harbor two LOV (light, oxygen, or voltage sensing) domains containing a flavin mononucleotide chromophore at their N-terminus and an AGCVIII (cAMP-dependent protein kinase A, cGMP-dependent protein kinase G, and phospholipid-dependent protein kinase C family subgroup VIII) serine/threonine kinase domain at their C-terminus.^2–4^ The model plant, *Arabidopsis thaliana* (Arabidopsis), has two phototropins, phot1 and phot2, that induce its phototropic responses.^2,5^ Phenotypic analysis of their mutants has revealed that phot1 is a highly sensitive photoreceptor that induces the phototropic responses of hypocotyls under blue light conditions at a fluence rate of 10^−5^ µmol m^–2^ s^–1^ or more, while phot2 is a lower sensitivity photoreceptor that induces the phototropic responses of hypocotyls at a fluence rate of 1 µmol m^–2^ s^–1^ or more.^5,6^ Both phot1 and phot2 bind to the NONPHOTOTROPIC HYPOCOTYL3 (NPH3) proteins which is required for an induction of phototropic response.^7–10^ NPH3 contains a broad complex, tramtrack, and bric-à-brac (BTB) domain at its N-terminus, the NPH3 domain in its mid-region, and a coiled-coil domain at its C-terminus.^11^ NPH3 exhibits some ubiquitin E3 ligase properties,^12^ but its biochemical functions during a phototropic response remain poorly understood.

NPH3 proteins are phosphorylated and localize at the plasma membrane under dark conditions. Due to blue light irradiation, those become transiently dephosphorylated and some part of those proteins are transiently released into the cytosol in a phot1-dependent manner.^6,13–15^ Recently, we identified seven phosphoserine (pS) residues, pS7 (or pS5), pS213, pS223, pS237, pS467, pS474 (or pS476), and pS722 (or pS723), on the NPH3 protein.^13^ These identified serine residues were all found to be phosphorylated in etiolated seedlings under dark conditions.^13^ Although each dephosphorylation of pS7 (or pS5), pS467, pS474 (or pS476), and pS722 (or pS723) by blue light irradiation has not been examined, the dephosphorylation of pS213, pS223, and pS237 in a phot1-activation-dependent manner was determined using anti-phospho-peptide antibodies.^15^ We created NPH3^SA^ and NPH3^SE^ mutants in which each of the seven serine residues, S7, S213, S223, S237, S467, S474, and S722, and three additional potentially phosphorylated serines, S5, S476, and S723, were substituted for alanine residues that cannot be phosphorylated or for glutamate residues that mimic a phosphorylated state. We then prepared transgenic *nph3* seedlings expressing *NPH3* wild-type (*NPH3*^WT^), NPH3^SA^, or *NPH3^SE^* genes (*35Spro:NPH3*^WT^ *nph3, 35Spro:NPH3*^SA^ *nph3*, and *35Spro:NPH3^SE^ nph3*, respectively).^13^

NPH3^SA^ proteins showed a shortened retention time in the cytosol, and the *35Spro:NPH3^SA^ nph3* seedlings showed reduced response of hypocotyl phototropism under blue light irradiation at the low fluence rate of 10^–5^ µmol m^–2^ s^–1^.^13^ In contrast, NPH3^SE^ proteins showed a lengthened retention time in the cytosol and the *35Spro:NPH3^SE^ nph3* seedlings did not show the response by blue light irradiation at fluence rates of 10^–3^ µmol m^–2^ s^–1^ or more.^13^ These findings were consistent with the phosphorylation status of NPH3 during phototropic responses, i.e., when blue light is irradiated at very low fluence rates (~10^−5^ µmol m^–2^ s^–1^), the activation level of phot1 is low and the dephosphorylation of NPH3 does not proceed, but at 10^−3^ µmol m^–2^ s^–1^ or more irradiation levels, the activation level of phot1 is high and the dephosphorylation of NPH3 is increased.^6,14,15^ Hence, we proposed a model in which the formation of a complex of phot1 and NPH3 on the plasma membrane reflects a steady state with regard to phot1 signaling whereas the dissociation of this complex represents an active state of phot1 signaling in hypocotyl phototropism.^13^ In this context, the lengthening of the retention time of NPH3^SE^ in the cytosol seemed to enhance phot1 signaling. This leads to an enhancement of phototropic responses under very low intensity blue light and a suppression of phototropic responses with a saturation of the phot1 signaling activity under high intensity blue light.^13^ In contrast, the shortening of the retention time of NPH3^SA^ in the cytosol seemed to suppress phot1 signaling, which leads to a suppression of the phototropic response under very low intensity blue light and a prevention of the saturation of the phot1 signaling activity to induce the phototropic responses under high intensity blue light.^13^

In contrast to phot1, the relationship between phot2 and NPH3 is still unclear. Phot2 autophosphorylates and induces phototropic responses in a phot1-independent manner under high-intensity blue light.^5,16^ Notably, however, the dissociation from the plasma membrane, aggregate formation, and dephosphorylation were not detected for NPH3 proteins in the *phot1* mutant under high-intensity blue light conditions.^6,14,15^ Another prior report indicated that phot2 suppresses the dissociation of NPH3 from the plasma membrane under high-intensity blue light.^10^ These results suggest that phot2-dependent phototropism does not require the dephosphorylation of NPH3 and its release from the plasma membrane to the cytosol, at least not at detectable levels.

We surmised that if the phosphomimetic NPH3^SE^ mutant functioned similarly to phosphorylated NPH3, the *35Spro:NPH3^SE^ phot1 nph3* transgenic lines would exhibit sufficient phototropic responses under blue light condition at 100 µmol m^–2^ s^–1^, as shown previously for *phot1* mutants.^16^ We further anticipated that if the NPH3^SA^ mutant negatively affected phot2 signaling activity, as reported in the case of phot1 signaling activity,^13^ and if phot2, unlike phot1, is insufficiently photosensitive to cause a saturation of its signaling activity even under blue light condition at 100 µmol m^–2^ s^–1^, the *35Spro:NPH3^SA^ phot1 nph3* transgenic lines would exhibit some decrease in their phototropic response under the same blue light conditions.

We here examined the phototropic responses of *phot1 nph3* double mutants expressing NPH3^WT^, NPH3^SA^, and NPH3^SE^ to further elucidate the role of NPH3 phosphorylation modifications in phot2 signaling. The transgenic lines,^13^ *35Spro:NPH3^WT^ nph3* #2, *35Spro:NPH3^SA^ nph3* #26, and *35Spro:NPH3^SE^ nph3* #7, were crossed with the *phot1* mutant (Salk_146058)^13^ to yield *35Spro:NPH3^WT^ phot1 nph3* #2, *35Spro:NPH3^SA^ phot1 nph3* #26, and *35Spro:NPH3^SE^ phot1 nph3* #7 lines. The induction of phototropic responses and measurements of hypocotyl curvatures were conducted as described previously,^16^ with some modifications. Two-day-old etiolated seedlings, which were grown along the surface of vertically oriented agar medium, were irradiated continuously with unilateral blue light. Because the phot2 blue light photoreceptor functions at high fluence rates of blue light (>1 μmol m^−2^ s^−1^), we used a fluence rate at 100 µmol m^–2^ s^–1^ in this study. The relationship between the hook position and the direction of the phototropic stimulus affects the phototropic curvature of Arabidopsis hypocotyls,^17^ and the phototropic responses of hypocotyls of the *35Spro:NPH3 phot1 nph3* seedlings were hardly induced by blue-light irradiation from the adaxial side of the hook (the cotyledon side) under this light condition. Therefore, the seedlings were classified into two subpopulations, one in which the hypocotyls were irradiated with blue light from the abaxial side of the hook and the other in which the hypocotyls were irradiated from the adaxial side of the hook, and the hypocotyl curvatures of the former were determined at the indicated time points (Figure 1).

**Figure 1.**
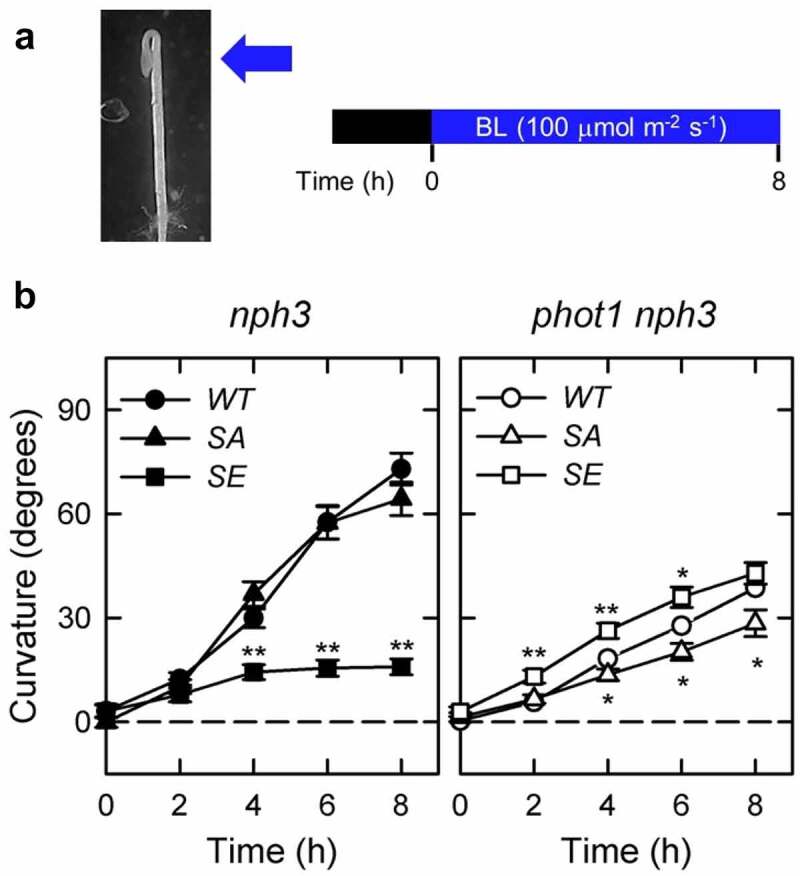
Effects of *NPH3^SA^* and *NPH3^SE^* mutations on the phototropic responses in the etiolated hypocotyls of *nph3* mutants and *phot1 nph3* double mutants. (a) Experimental scheme of the investigation of hypocotyl phototropism. Two-day-old etiolated seedlings were irradiated continuously with blue light (BL) at 100 µmol m^–2^ s^–1^ from the abaxial side of the hook. (b) Time courses of the phototropic responses in the *35Spro:NPH3^WT^ nph3* #2 (*WT* in the left panel), *35Spro:NPH3^SA^ nph3* #26 (*SA* in the left panel), *35Spro:NPH3^SE^ nph3* #7 (*SE* in the left panel), *35Spro:NPH3^WT^ phot1 nph3* #2 (*WT* in the right panel), *35Spro:NPH3^SA^ phot1 nph3* #26 (*SA* in the right panel), and *35Spro:NPH3^SE^ phot1 nph3* #7 (*SE* in the right panel). The data shown are the mean values ± SE from 18–40 seedlings. Asterisks indicate statistically significant differences from the curvatures of *WT* (*p < 0.05, **p < 0.01).

Two-day-old etiolated hypocotyls of the *35Spro:NPH3^SA^ nph3* #26 seedlings showed the same degree of hypocotyl curvatures as *35Spro:NPH3^WT^ nph3* #2, although the *35Spro:NPH3^SE^ nph3* #7 line showed decreased hypocotyl curvatures in comparison with the *35Spro:NPH3^WT^ nph3* #2 under unilateral blue light at 100 µmol m^–2^ s^–1^ (Figure 1b), as described previously.^13^ By contrast, the *35Spro:NPH3^SA^ phot1 nph3* #26 line showed reduced hypocotyl curvatures compared to *35Spro:NPH3^WT^ phot1 nph3* #2, whereas the *35Spro:NPH3^SE^ phot1 nph3* #7 transgenic line showed increased hypocotyl curvatures compared to *35Spro:NPH3^WT^ phot1 nph3* #2 (Figure 1b). These data indicated that NPH3^SE^, but not NPH3^SA^, is suitable for phot2-dependent phototropism under unilateral blue light at 100 µmol m^–2^ s^–1^. These findings also suggest that the dephosphorylation of NPH3 is not required for phot2-dependent phototropism in *phot1* mutants but instead causes a suppression of phot2-dependent phototropism, at least under blue light condition at 100 µmol m^–2^ s^–1^.

To next determine the subcellular localization of NPH3^SA^ and NPH3^SE^ in the *phot1* mutants, the transgenic lines,^13^ *35Spro:YFP-NPH3^SA^ nph3* and *35Spro:YFP-NPH3^SE^ nph3*, were crossed with the *phot1* mutants to generate the *35Spro:NPH3^SA^ phot1 nph3* and *35Spro:NPH3^SE^ phot1 nph3* lines. It has already been demonstrated in a previous study that both of these *nph3* transgenic lines expressing YFP-tagged NPH3^SA^ and NPH3^SE^ display similar phototropic responses to those expressing NPH3^SA^ and NPH3^SE^ with no YFP tag.^13^ In our current experiments, we analyzed the subcellular localization of YFP fluorescence in the etiolated hypocotyls of these lines after blue light irradiation at 100 µmol m^–2^ s^–1^ for 6 hours. Both YFP-NPH3^SA^ and YFP-NPH3^SE^ localized at the plasma membrane and did not dissociate under this light condition (Figure 2), as found previously for YFP-NPH3^WT^ in *phot1* mutants.^6^ This indicated that the mutations leading to unphosphorylatable and phosphomimetic NPH3 proteins have different effects on phot2-dependent hypocotyl phototropism without a difference of their subcellular localization.

**Figure 2.**
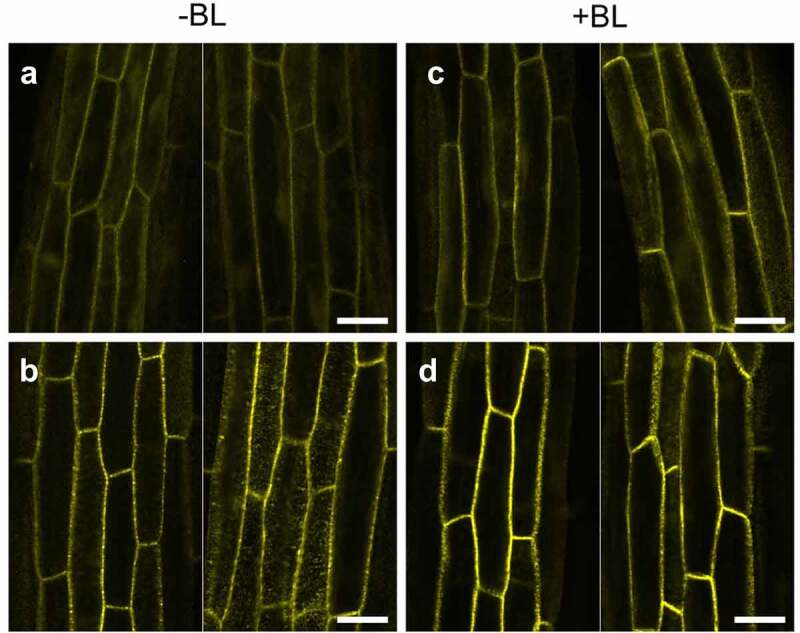
Localization pattern changes for YFP-NPH3 proteins in the *phot1 nph3* mutants in response to blue light irradiation. Two-day-old etiolated seedlings of the *35Spro:YFP-NPH3^SA^ phot1 nph3* (a, c) and *35Spro:YFP-NPH3^SE^ phot1 nph3* (b, d) lines were irradiated (c, d: +BL) or not (a, b: -BL) with blue light at 100 µmol m^–2^ s^–1^ for 6 h. YFP signals were detected under a TCS-SP5 confocal laser scanning microscope (Leica Microsystems), as described previously.^13^ Two representative images are shown. White bar, 25 µm.

Our current study revealed that the phosphomimetic NPH3^SE^ functions well in phot2-dependent phototropism, whereas the unphosphorylatable NPH3^SA^ cannot do so, under blue light condition at 100 µmol m^–2^ s^–1^. This observation is not inconsistent with their effects on phot1-dependent phototropism. As proposed previously,^13^ the mutations that generate the phosphomimetic NPH3^SE^ probably enhance phot signaling and thereby promote phot2-dependent phototropism, but also suppress the phot1-dependent phototropic response through a saturation of phot1 signaling activity under high intensity blue light conditions. The mutations that produce the unphosphorylatable NPH3^SA^ protein likely suppress phot signaling, and therefore downregulate phot2-dependent phototropism, but increase phot1-dependent phototropism through a photosensory adaptation to the hyperactivation of phot1 by high-intensity blue light. The activation of phot1, but not phot2, leads to the dephosphorylation of NPH3, suggesting that phot1 negatively controls phot2 signaling and that the hyperactivation of both phot1-NPH3 and phot2-NPH3 complexes does not occur at the same time under high-intensity blue light conditions.

The question of how the phosphorylation status of NPH3 affects phot2-dependent phototropism remains. Although phot2 has no capacity, or lacks the activation ability, to induce the release of NPH3 to the cytosol even under blue light condition at 100 µmol m^–2^ s^–1^, the activation of phot2 may induce the dissociation of phot2-NPH3 complexes at the plasma membrane surface without the release of NPH3 to the cytosol. A previous study has also indicated that NPH3^SA^ and NPH3^SE^ have different effects on phot1-dependent phototropism without any detectable differences in their subcellular localizations under blue light condition at 1.7 × 10^−3^ µmol m^–2^ s^–1^.^13^ The dynamics of phot2-NPH3 complex formation on the plasma membrane will need to be examined during the phototropic response under high-intensity blue light conditions in future studies.
